# No detectable alloreactive transcriptional responses under standard sample preparation conditions during donor-multiplexed single-cell RNA sequencing of peripheral blood mononuclear cells

**DOI:** 10.1186/s12915-020-00941-x

**Published:** 2021-01-20

**Authors:** Christopher S. McGinnis, David A. Siegel, Guorui Xie, George Hartoularos, Mars Stone, Chun J. Ye, Zev J. Gartner, Nadia R. Roan, Sulggi A. Lee

**Affiliations:** 1 Department of Pharmaceutical Chemistry, University of California San Francisco, San Francisco, CA USA; 2 Department of Medicine, Division of HIV/AIDS, UCSF, San Francisco, CA USA; 3 Gladstone Institute of Virology, San Francisco, CA USA; 4 Department of Urology, UCSF, San Francisco, CA USA; 5 Institute for Human Genetics, UCSF, San Francisco, CA USA; 6 Graduate Program in Biological and Medical Informatics, UCSF, San Francisco, CA USA; 7 Department of Laboratory Medicine, UCSF, San Francisco, CA USA; 8 Vitalant Research Institute, UCSF, San Francisco, CA USA; 9 Department of Epidemiology and Biostatistics, UCSF, San Francisco, CA USA; 10 Department of Bioengineering and Therapeutic Sciences, UCSF, San Francisco, CA USA; 11 Parker Institute for Cancer Immunotherapy, San Francisco, CA USA; 12 Chan Zuckerberg BioHub, UCSF, San Francisco, CA USA; 13 Center for Cellular Construction, UCSF, San Francisco, CA USA; 14 Helen Diller Family Comprehensive Cancer Center, San Francisco, CA USA

**Keywords:** scRNA-seq, Sample multiplexing, Sample preparation, Alloreactivity, PBMCs

## Abstract

**Background:**

Single-cell RNA sequencing (scRNA-seq) provides high-dimensional measurements of transcript counts in individual cells. However, high assay costs and artifacts associated with analyzing samples across multiple sequencing runs limit the study of large numbers of samples. Sample multiplexing technologies such as MULTI-seq and antibody hashing using single-cell multiplexing kit (SCMK) reagents (BD Biosciences) use sample-specific sequence tags to enable individual samples to be sequenced in a pooled format, markedly lowering per-sample processing and sequencing costs while minimizing technical artifacts. Critically, however, pooling samples could introduce new artifacts, partially negating the benefits of sample multiplexing. In particular, no study to date has evaluated whether pooling peripheral blood mononuclear cells (PBMCs) from unrelated donors under standard scRNA-seq sample preparation conditions (e.g., 30 min co-incubation at 4 °C) results in significant changes in gene expression resulting from alloreactivity (i.e., response to non-self). The ability to demonstrate minimal to no alloreactivity is crucial to avoid confounded data analyses, particularly for cross-sectional studies evaluating changes in immunologic gene signatures.

**Results:**

Here, we applied the 10x Genomics scRNA-seq platform to MULTI-seq and/or SCMK-labeled PBMCs from a single donor with and without pooling with PBMCs from unrelated donors for 30 min at 4 °C. We did not detect any alloreactivity signal between mixed and unmixed PBMCs across a variety of metrics, including alloreactivity marker gene expression in CD4+ T cells, cell type proportion shifts, and global gene expression profile comparisons using Gene Set Enrichment Analysis and Jensen-Shannon Divergence. These results were additionally mirrored in publicly-available scRNA-seq data generated using a similar experimental design. Moreover, we identified confounding gene expression signatures linked to PBMC preparation method (e.g., Trima apheresis), as well as SCMK sample classification biases against activated CD4+ T cells which were recapitulated in two other SCMK-incorporating scRNA-seq datasets.

**Conclusions:**

We demonstrate that (i) mixing PBMCs from unrelated donors under standard scRNA-seq sample preparation conditions (e.g., 30 min co-incubation at 4 °C) does not cause an allogeneic response, and (ii) that Trima apheresis and PBMC sample multiplexing using SCMK reagents can introduce undesirable technical artifacts into scRNA-seq data. Collectively, these observations establish important benchmarks for future cross-sectional immunological scRNA-seq experiments.

**Supplementary information:**

**Supplementary information** accompanies this paper at 10.1186/s12915-020-00941-x.

## Background

Recent advances in single-cell RNA sequencing (scRNA-seq) technologies have dramatically increased assay throughput from ~ 10^2^ to 10^4^–10^6^ cells per experiment [1]. However, many applications of scRNA-seq workflows (e.g., 10x Genomics) require individual samples to be processed in parallel, which translates to prohibitively-high assay costs for population-scale studies requiring large numbers of samples. Several scRNA-seq sample multiplexing techniques have been developed which enable users to circumvent this limitation by processing samples in a pooled format [2–12]. By avoiding the usual requirement for processing distinct samples individually, these technologies increase scRNA-seq cell and sample throughput while minimizing technical confounders (e.g., doublets and batch effects). Two main types of sample multiplexing approaches have been described: (i) in silico genotyping using natural [7–10] or artificial [11, 12] genomic variants and (ii) tagging cell membranes with sample-specific DNA barcodes using lipid-modified oligonucleotides (LMOs; e.g., MULTI-seq) [2], DNA-conjugated antibodies [3–5] (e.g., BD single-cell multiplexing kit (SCMK) [5]), or methyltetrazine-modified DNA “ClickTags” [6]). Despite the increasing popularity of sample multiplexing, benchmarking studies aiming to measure transcriptional changes induced by mixing cell suspensions during scRNA-seq sample preparation have not been described. Determining the extent to which these changes might occur is critical, as they would confound cross-sectional data interpretation.

Mixing-specific transcriptional responses could occur when peripheral blood mononuclear cells (PBMCs) from unrelated donors are pooled during scRNA-seq sample preparation. Co-culturing human leukocyte antigen (HLA) mismatched PBMCs causes a rapid and potent allogeneic response wherein T lymphocytes are stimulated through T cell receptor binding to “non-self” major and minor histocompatibility complex proteins [13–16]. For example, CD154+ alloreactive CD4+ T cells were detected within 2 h after HLA-mismatched lymphocyte mixing [13], while bulk transcriptomics identified a ~ 5-fold increase within 24 h of alloreactivity-associated gene expression relative to HLA-matched lymphocytes [14]. Although pooled samples are maintained on ice for short durations during scRNA-seq sample preparation, it is unclear whether the allogeneic response may occur at low temperatures or whether transient periods of warming (e.g., during droplet emulsion at room temperature) are sufficient to drive alloreactivity. Considering that scRNA-seq is sensitive to transcriptional responses in rare cell sub-populations which are obscured by bulk assays, directly assessing whether alloreactivity will confound downstream scRNA-seq analyses is a critical benchmark for large-scale immunological studies [17].

Here, we performed scRNA-seq using the 10x Genomics platform on PBMC samples isolated from eight unrelated healthy donors pooled under conditions where cells from a single donor were processed in isolation or after donor pooling. Donor identities for each cell were assigned using SCMK and MULTI-seq data, as well as the in silico genotyping pipeline, souporcell [8]. We observed cell-type biases among SCMK classification results which were not due to sub-optimal antibody labeling conditions or the presence of MULTI-seq LMOs. We additionally did not observe robust, mixing-associated changes in PBMC cell type frequencies, global transcriptional profiles, or alloreactivity-associated gene expression in any PBMC cell type. Finally, we validated the observed lack of alloreactivity in a publicly-available scRNA-seq dataset where PBMCs from two unrelated donors were sequenced in isolation and after pooling [18]. As a result, we conclude that pooling PBMCs from unrelated donors under standard 10x Genomics-based scRNA-seq sample preparation conditions (e.g., 30 min co-incubation at 4 °C) does not result in any detectable alloreactivity at the RNA level.

### Study design

**Fig. 1:**
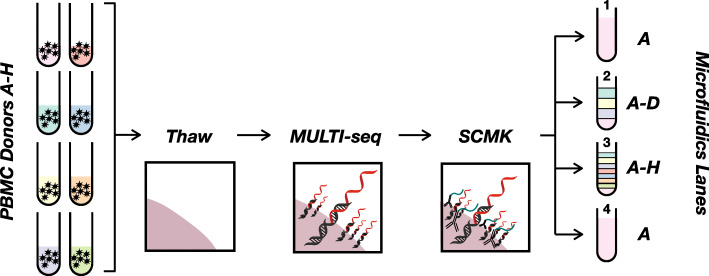
Schematic overview of experimental design. PBMCs from 8 healthy HLA-mismatched donors (tubes on left) were barcoded with MULTI-seq LMOs (black double-helix hybridized to red DNA barcode) and BD single-cell multiplexing kit (SCMK) antibodies (black antibody conjugated to teal DNA barcode). Cells were then strategically pooled to directly assess whether mixing HLA-mismatched PBMCs during scRNA-seq causes alloreactivity

To assess whether mixing PBMCs from unrelated donors causes alloreactivity during scRNA-seq, we performed a cross-sectional study of PBMCs isolated from 8 unrelated healthy donors (Fig. 1; Experimental methods). To record the donor-of-origin for each cell, PBMC samples were tagged with donor-specific MULTI-seq [2] and/or SCMK antibody-DNA [5] barcodes. PBMCs were mixed for 30 min at 4 °C prior to emulsion across four droplet microfluidics lanes (10x Genomics) at room temperature. The 30-min pooled incubation was chosen to mimic the typical processing time required for preparing samples for multiplexed scRNA-seq analysis. Following scRNA-seq data pre-processing, quality-control, cell type annotation, and sample demultiplexing (Computational Methods), we compared the expression profile of unmixed donor A PBMCs (microfluidic lane #1) to donor A PBMCs mixed with donors B-D (microfluidic lane #2), donors B-H (microfluidic lane #3), and an unmixed donor A PBMC technical replicate prepared without antibody-DNA labeling (microfluidic lane #4). We hypothesized that if co-incubation of PBMCs from unrelated donors for 30 min at 4 °C causes detectable alloreactivity, then mixed and unmixed donor A PBMCs would exhibit more variable gene expression profiles than what is observed due to technical variation.

## Results

### MULTI-seq classifies PBMCs more accurately than SCMK

**Fig. 2:**
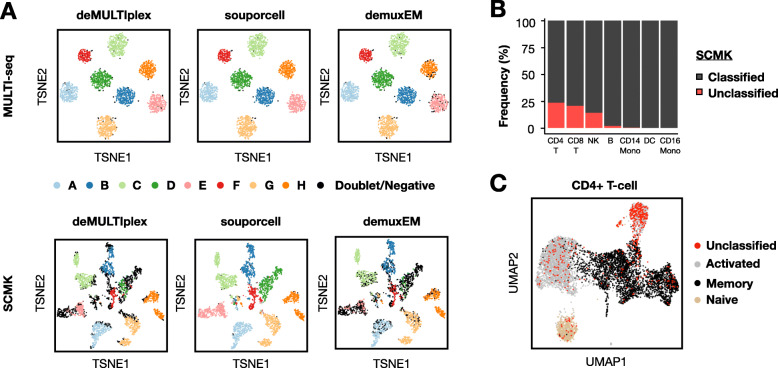
MULTI-seq and SCMK classifications largely match in silico genotyping, with lower SCMK classification efficiency and bias against activated CD4+ T cells. **a** Sample classification results from three demultiplexing pipelines (e.g., deMULTIplex, souporcell, and demuxEM) projected onto MULTI-seq (top) and SCMK (bottom) sample barcode space. *n* = 4032 cells from microfluidic lane #3. **b** Classification frequencies across all PBMC cell types following SCMK sample demultiplexing. **c** SCMK unclassified cells in CD4+ T cell gene expression space. *n* = 6879 CD4+ T cells

We first assessed the performance of MULTI-seq and SCMK by comparing the results of three distinct demultiplexing workflows on donor A-H PBMCs from microfluidic lane #3: (i) deMULTIplex, (ii) demuxEM, and (iii) souporcell. deMULTIplex [2] and demuxEM [4] are algorithms that function on sample barcode count matrices, while souporcell is an in silico genotyping pipeline that functions on gene expression data [8]. MULTI-seq and SCMK classifications were largely consistent with souporcell (Fig. 2a)—e.g., among cells classified as donors A-H using souporcell, 99.9% and 99.0% of donor classifications were consistent for MULTI-seq and SCMK, respectively. However, while 1.5% of cells remained unclassified following MULTI-seq demultiplexing, 36.2% of cells remained unclassified after SCMK demultiplexing. This decrease in classification efficiency was also observed when compared to the demuxEM results (Table 1).

**Table 1:** MULTI-seq and SCMK classification performance summary

Method	MULTI-seq	SCMK
**% Unclassified (deMULTIplex)**	1.5%	36.2%
**% Unclassified (demuxEM)**	2.8%	35.4%
**% souporcell Donor Match (deMULTIplex)**	99.9%	99.0%
**% souporcell Donor Match (demuxEM)**	98.8%	97.7%

Classification performance was determined using two statistics: (i) % Unclassified, proportion of cells classified into donor groups by souporcell that remain unclassified by MULTI-seq or SCMK; (ii) % Donor Match, proportion of cells classified into the same donor group by MULTI-seq or SCMK and souporcell. Both metrics were computed using results from both deMULTIplex and demuxEM

To assess whether cells that remained unclassified following SCMK demultiplexing were randomly distributed throughout the scRNA-seq data, we computed the frequency of unclassified cells for each PBMC cell type. This analysis revealed that T lymphocytes and NK cells were especially likely to remain unclassified in SCMK data (Fig. 2b). Moreover, activated CD4+ T cells were particularly prominent among the unclassified CD4+ T cells (Fig. 2c).

It is conceivable that the presence of LMOs and/or sub-optimal SCMK labeling buffer conditions caused the observed classification biases in PBMCs. To address whether LMOs interfere with SCMK labeling, we generated scRNA-seq data where cells from 7 PBMC donors were pooled after labeling with SCMK reagents but not LMOs. As was observed previously, SCMK classifications were similarly biased against T lymphocytes and NK cells (Additional file 1: Figs. S1A, S1D), with activated CD4+ T cells being particularly difficult to classify (Additional file 1: Figs. S1B, S1E). To address whether SCMK classification biases in PBMCs is due to sub-optimal antibody labeling conditions, we determined the extent of classification bias in a publicly-available scRNA-seq dataset provided by the SCMK reagent supplier where PBMCs were cultured in vitro for 24 h in the presence or absence of anti-CD3/anti-CD28 antibodies [19]. In these data, SCMK classifications were biased against T lymphocytes and NK cells, despite optimal SCMK labeling conditions (Figs. S1C, F). Collectively, these results illustrate that SCMK reagents produce biased classifications when applied to PBMCs. For these reasons, MULTI-seq donor classifications were used for all subsequent gene expression analyses.

### Trima apheresis introduces biologically-relevant confounders into PBMC scRNA-seq data

The PBMCs that were used in this study came from whole blood that was processed using Ficoll-Paque density gradient centrifugation. Notably, these samples either underwent (donors D–H) or did not undergo (donors A–C) apheresis using Trima filtration, a method to enhance leukocyte yield during sample preparation [20, 21]. Initial inspection of MULTI-seq donor classifications revealed that PBMCs predominantly clustered according to the processing method—e.g., Trima vs. Ficoll (Additional file 1: Fig. S2A). Upon sub-clustering CD14+ classical monocytes and natural killer (NK) cells, we observed that Trima and Ficoll classical monocytes expressed variable levels of the histone component gene HIST1H1C, as well as two genes involved in monocyte differentiation, MNDA and CEBPB (Additional file 1: Fig. S2B, left) [22]. Moreover, we observed that Trima and Ficoll NK cells differentially expressed the immune cytokine IFNG, cytolytic genes GZMA and PRF1, and the stress marker JUN (Additional file 1: Fig. S2B, right) [23]. These results suggest that apheresis using Trima filters induces confounding changes in gene expression patterns associated with differentiation state, cytolytic activity, and stress across multiple PBMC cell types. These signatures are consistent with prior observations [24] and should be accounted for in future analyses. Thus, to avoid these confounding effects when comparing donor- and mixing-specific expression profiles, we restricted our subsequent analyses to PBMC samples processed without Trima filtration.

### Mixing PBMCs from unrelated healthy donors during scRNA-seq sample preparation does not cause a detectable allogeneic transcriptional response

**Fig. 3:**
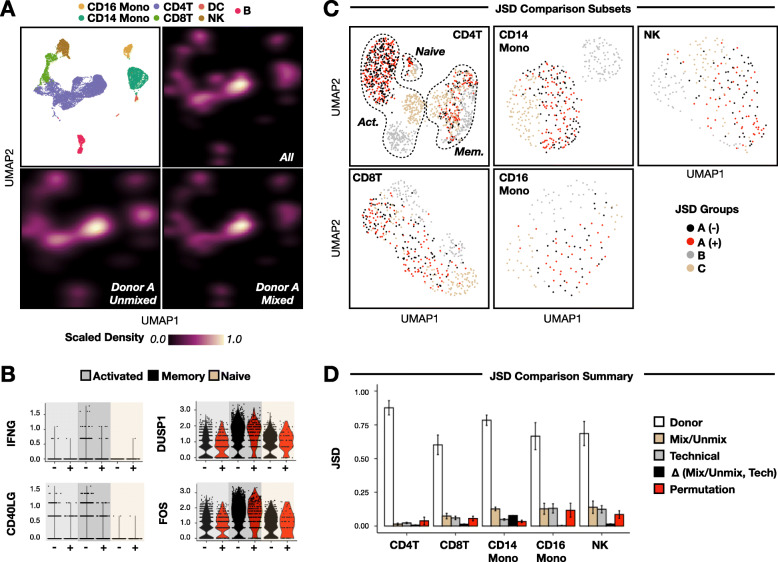
Mixing HLA-mismatched PBMCs does not cause allogenic response during multiplexed scRNA-seq sample preparation. **a** Sample classification results plotted as densities in PBMC gene expression space (top left) grouped according to unmixed donor A PBMCs (bottom left), mixed Donor A PBMCs (bottom right), and Donors A-C PBMCs (top right). **b** Expression of genes known to be upregulated (e.g., IFNG and CD40LG) or downregulated (e.g., DUSP1 and FOS) by CD4+ T lymphocytes during an allogenic response across unmixed (black) and mixed (red) donor A CD4+ T cell subsets. **c** Representative CD4+ T cell, CD8+ T cell, CD14+ monocyte, CD16+ monocyte, and NK cell gene expression embeddings following iterative subsetting to select equal numbers from each JSD comparison group. Cells are colored according to donor ID (e.g., **a**, **b**, **c**) and mixing status (e.g., − = unmixed, + = mixed). *n* = 1336 CD4+ T cells, 448 CD8+ T cells, 864 CD14+ monocytes, 136 CD16+ monocytes, and 224 NK cells. **d** JSD analysis summary. Bar plots denote average JSD between PBMC donors (white), mixed/unmixed donor A cells (beige), technical replicates (gray), and donor A cells following label permutation (red). Difference in JSD scores between mixed/unmixed and technical replicates depicted in black. Error bars denote +/− 1 standard deviation. *n* = 100 iterations

To assess whether mixing PBMCs from unrelated donors induces alloreactivity during multiplexed scRNA-seq, we compared the expression profiles of mixed and unmixed donor A PBMCs. Mapping the densities of mixed and unmixed donor A sample classifications onto PBMC gene expression space (Fig. 3a, top left) did not reveal any qualitative shifts in global gene expression profiles (Fig. 3a, bottom). Notably, such shifts in classification densities were observed when including PBMCs from donors B and C (Fig. 3a, top right), suggesting that natural inter-donor variation is more pronounced than intra-donor variation due to PBMC mixing. Indeed, PBMC cell-type frequencies were similarly-variable between donors, while no statistically-significant shifts in cell-type frequencies were linked to mixing status (Additional file 1: Fig. S3A, S3B).

Next, we focused on CD4+ T cells because of their known involvement in alloreactivity [13–16]. Mixed and unmixed donor A CD4+ T cells expressed genes known to be involved in an allogeneic response [13–16] at similar levels (Fig. 3b). Similar to the full dataset, mixed and unmixed donor A cells were clustered together in CD4+ T cell gene expression space (Fig. 3c, top left). Finally, no statistically-significant shifts in CD4+ T cell subtype frequencies were linked to mixing status (Additional file 1: Fig. S3C, S3D).

Expanding our analysis to other PBMC cell types, we next applied two unbiased approaches to measure any putative allogeneic response signature. First, we used the dissimilarity metric Jensen-Shannon Divergence (JSD) [25] to compute sample-level differences for each PBMC cell type. To control for differences in cell type proportions, we randomly subsetted equal numbers of each cell type from each experimental group during PBMC sub-clustering (Fig. 3c) and repeated this workflow 100 times. Across the 100 iterations, we then computed the average JSD scores between donors, unmixed and mixed donor A cells, and technical replicates (Computational methods). For all cell types, inter-donor JSD scores were greater than those linked to mixing status and technical replicate, while mixing status JSD scores were greater than technical replicate JSD scores for CD8+ T cells, CD14+ monocytes, and NK cells (Fig. 3d).

To determine the likelihood of observing elevated mixing status JSD scores relative to technical replicates by chance, we repeated this workflow after permuting donor A classifications. Specifically, we reasoned that if permuted JSD scores were greater than the difference between observed mixing status and technical replicate JSD scores, then the observed differences are not significant. To this end, JSD scores after donor A label permutation were larger than the experimental JSD score differential in all cell types except CD14+ classical monocytes (Fig. 3c).

The second unbiased approach we utilized to look for allogeneic response signatures was Gene Set Enrichment Analysis (GSEA) [26, 27]. Specifically, we applied GSEA to donor A cells from each PBMC cell type to determine whether pathways involved in immune activation and/or alloreactivity were enriched in mixed relative to unmixed cells. This analysis revealed that among unmixed donor A cells, activated CD4+ T cells were enriched for humoral immune response genes and dendritic cells were enriched for epigenetic regulation and cell killing (Additional file 2: Supplemental Table 1). Notably, these detected gene sets in unmixed cells are not consistent with an allogeneic response, and no enriched gene sets were identified among any mixed donor A cell types.

It is conceivable that the presence of LMOs and antibody-DNA barcodes could delay or block any allogeneic response between PBMCs from unrelated donors. To explore this possibility, we repeated our analytical workflow on a publicly-available scRNA-seq dataset where PBMCs from two unrelated healthy donors were sequenced in isolation and after pooling and incubation on ice for 30 min [18]. Mirroring our previous observations, mixing was not robustly associated with any statistically-significant shifts in PBMC cell type proportions (Additional file 1: Fig. S4A, S4B) or CD4+ T cell subtypes (Additional file 1: Fig. S4C, S4D). Moreover, cells clustered primarily by donor and not mixing status (Additional file 1: Fig. S4E), and inter-donor JSD scores were greater than mixing status JSD scores for all cell types (Additional file 1: Fig. S4F). Although this experimental design did not allow comparisons between JSD scores linked to mixing status and technical replicates, permuted and mixing status JSD scores were on-par for most cell types (including CD14+ monocytes; Additional file 1: Fig. S4F). Finally, while GSEA identified a number of enriched gene sets among mixed PBMCs in these data (e.g., protein trafficking, translation, non-sense mediated decay, viral gene expression, and amino acid metabolism; Additional file 2: Supplemental Table 2), these gene sets were unrelated to alloreactivity and were shared across most PBMC cell types, suggesting they were caused by batch effects between the mixed and unmixed scRNA-seq libraries.

Collectively, these targeted and unbiased quantitative comparisons across all PBMC cell types in two, independently-generated scRNA-seq datasets demonstrate that mixing PBMCs from unrelated donors under standard multiplexed scRNA-seq sample preparation conditions (e.g., 30 min co-incubation at 4 °C) does not result in a detectable allogeneic transcriptional response.

## Discussion

Sample multiplexing approaches for scRNA-seq are being increasingly utilized by the single-cell genomics field to reduce assay costs while improving data breadth and quality. However, the impact of pooling PBMCs from unrelated donors during scRNA-seq sample preparation on gene expression patterns has not yet been adequately quantified. Here, we used the 10x Genomics scRNA-seq platform to directly compare the gene expression profiles of PBMCs prepared for sequencing alone or after mixing with PBMCs from unrelated donors for 30 min at 4 °C. We found no evidence of global changes in gene expression profiles in any PBMC cell type (quantified using JSD and GSEA), PBMC cell type proportions, or alloreactivity marker gene expression in CD4+ T cells linked to PBMC mixing status. Although PBMCs actively participating in an allogeneic response were not included in this study, these observations were mirrored in an independently-generated, publicly-available PBMC scRNA-seq dataset [18], demonstrating that mixing unrelated PBMCs during sample-multiplexed scRNA-seq sample preparation does not result in a detectable allogeneic response. Notably, it is possible that cellular responses to pooling could be detected by assays measuring levels of biological information with faster regulatory kinetics (e.g., cell surface protein assays [28, 29]) or under different scRNA-seq experimental conditions (longer periods of co-incubation at higher temperatures, mixing cells from distinct species, etc.). To this end, the experimental design employed in this study can be used to benchmark the prevalence of sample mixing-specific confounders in future single-cell genomics experiments.

In addition to the alloreactivity analysis, we found that Trima apheresis can introduce confounding variables into scRNA-seq data, which suggests that this PBMC preparation method should be avoided in future experiments. Moreover, we found that SCMK demultiplexing results were biased against activated CD4+ T cells and other lymphoid cell types. This observation was mirrored in two scRNA-seq datasets generated (i) in the absence of LMOs and (ii) with optimized SCMK antibody-DNA labeling conditions. These findings are in contrast to the original Cell Hashing report [3], where PBMCs were systematically demultiplexed following incubation with a panel of DNA-conjugated antibodies selected for their uniform targeting of all known PBMC cell populations. Notably, the exact antigens targeted by the commercial SCMK reagents used in this study are proprietary and unknown, but our findings suggest that the “universal” antigens targeted by these antibody-DNA conjugates may be differentially-expressed by distinct cell types in ways that interfere with sample classification. It remains to be determined whether “universal” Cell Hashing reagents from BioLegend, which target human beta-2-microglobulin and CD298, suffer from similar performance issues. Thus, users should exercise caution before using SCMK reagents, for example by testing the uniformity of antibody binding conducting flow-cytometry experiments with fluorophore-conjugated DNA probes that hybridize to SCMK oligonucleotide domains. In any case, validation of surface antigen expression across all cells in a given experimental system and/or careful data quality-control is necessary to avoid systematically-biased interpretations.

## Conclusion

Collectively, this study proposes three critical benchmarks for future sample-multiplexed scRNA-seq analyses of PBMCs. First, we demonstrate that alloreactivity can be disregarded as a potential confounder when analyzing scRNA-seq data from PBMCs of unrelated donors pooled under standard multiplexed scRNA-seq sample preparation conditions. These conclusions may not, however, be generalizable to all single-cell genomics assays or sample preparation workflows. Second, we demonstrate that Trima apheresis of PBMCs introduces artifactual gene expression signatures which can confound downstream scRNA-seq data analyses. Third, we demonstrate that SCMK reagents are biased against certain PBMC cell types, which illustrates the importance of validating antibody-based sample multiplexing technology performance.

## Experimental methods

### scRNA-seq sample preparation, 8-donor MULTI-seq/SCMK PBMC experiment

PBMCs were provided by the Vitalant Research Institute. PBMCs were thawed at 37 °C and washed one time with warm media (RPMI (Corning, Cat#10-040-CV), supplemented with 10% FBS (VWR, Cat#97068-085) and Benzonase (1:1000, Sigma-Aldrich, Cat#E1014)) and one time with 2% FBS in PBS (Ca^++^ and Mg^+^ free, Corning, Cat#21-031-CV) before counting cells (Nexcelom K2). Live cells were then enriched using a dead-cell removal kit (STEM Cell, Cat#17899). Live cells were then washed with PBS and labeled with LMOs, as described previously [2]. LMOs were then quenched while washing cells with 1% BSA in cold PBS. Cells were then incubated with 5ul human Fc Block with 95ul 2% FBS in PBS at 4 °C for 15 min before staining with SCMK and AbSeq antibodies (BD Biosciences) at 4 °C for 60 min. Notably, AbSeq data was not analyzed in this study, and a subset of donor A PBMCs were not labeled with antibody-DNA conjugates (sequenced in microfluidics lane 4). Cells were then washed twice by using 0.04% BSA (non-acetylate, Sigma-Aldrich; B6917) in cold media before incubation for 30 min at 4 °C either alone (e.g., donor A) or in a pooled format (e.g., donors A–D or A–H). Cell viabilities for each donor prior to pooling ranged from 89 to 97%. Finally, cells were isolated via droplet emulsion across four 10x Genomics microfluidic lanes (V2) to yield 5000 cells.

### scRNA-seq sample preparation, 7-donor SCMK PBMC experiment

Healthy donor PBMCs were used from the ImmVar project [30], isolated from whole blood, and frozen as described therein. Vials from 7 patients, each with 1 million, were thawed at 37 °C and washed once with warm media before staining with SCMK antibodies. Briefly, cells were stained for 20 min at room temperature before being washed 3 times in 2 mL BD stain buffer. Cells were then counted, pooled, resuspended in 0.04% BSA in PBS, and isolated via droplet emulsion across a single 10x Genomics microfluidic lane (V2) to yield 50,000 cells.

### Next-generation sequencing and library preparation

cDNA expression, MULTI-seq, and SCMK libraries were prepared as described previously [2] or according to supplier recommendations. Notably, following size-selection of MULTI-seq and SCMK oligos after cDNA amplification, two separate sample-index PCRs were performed for the MULTI-seq and SCMK oligos using separate i7 indices. For the 8-donor and 7-donor PBMC experiments, cDNA expression and SCMK libraries were pooled and sequenced on a single NovaSeq 6000 lane (one lane per experiment). MULTI-seq libraries were sequenced separately using the MiSeq (V3).

## Computational methods

### Data pre-processing

Eight next-generation sequencing libraries from four separate experiments were analyzed in this study. Data pre-processing details for each library are summarized below:

**Table:** 

Experiment	Library	Details
8-Donor PBMC	scRNA-seq	Cell Ranger (v3.0.0), hg19 reference, read-depth normalization. In silico *genotyping* using souporcell [8]
8-Donor PBMC	MULTI-seq	deMULTIplex (v1.0.2), Hamming Distance = 1
8-Donor PBMC	SCMK	deMULTIplex (v1.0.2), Hamming Distance = 5
7-Donor PBMC	scRNA-seq	Cell Ranger (v3.0.0), custom hg19 reference containing SCMK barcodes. In silico genotyping using Demuxlet [7] (genotype error offset = 0.1, alpha = 0.0, 0.5, mapping quality = 255)
7-Donor PBMC	SCMK	Cell Ranger (v3.0.0) custom hg19 reference containing SCMK barcodes. R2 FASTQs trimmed using Trimmomatic [31] (single-end mode, HEADCROP = 25, CROP = 45)
Zheng et al. PBMC	scRNA-seq	Cell Ranger (v3.0.0), hg19 reference, read-depth normalization
2-Condition PBMC (BD)	scRNA-seq	Downloaded from provider [19]
2-Condition PBMC (BD)	SCMK	Downloaded from provider [19]

Notably, because the MULTI-seq and SCMK barcode sequences are 8 and 40 nucleotides in length, respectively, the Hamming Distance alignment threshold applied to SCMK data was increased to 5 (default = 1) to account for the increased probability of random sequencing errors.

### Data quality-control

The same quality-control workflows were applied to the 8-donor (Additional file 1: Fig. S5) and Zheng et al. (Additional file 1: Fig. S6) PBMC datasets using Seurat [32, 33]. First, cells with fewer than 250 RNA UMIs and genes with fewer than 3 UMIs across all cells were discarded. These parsed datasets were then normalized using “SCTransform” prior to unsupervised clustering and dimensionality reduction using PCA and UMAP. Low-quality cells selected via membership in clusters associated with low total RNA UMIs and/or high proportions of mitochondrial gene expression were then removed (Additional file 1: Fig. S5A, S6A).

Next, we split the cleaned datasets by microfluidic lane-of-origin and identified heterotypic doublets using DoubletFinder [34]. Notably, DoubletFinder was run on each lane independently to ensure that representative artificial doublets were constructed for each lane (e.g., multi-donor doublets were not generated for the unmixed data subsets). Moreover, we did not use MULTI-seq, SCMK, or souporcell classification results for doublet detection because each approach would produce different results for each lane (e.g., no doublets would be detected for single-donor datasets). DoubletFinder resulted in the removal of 1287 and 1832 heterotypic doublets in the 8-donor PBMC (Additional file 1: Fig. S5B) and Zheng et al. PBMC (Additional file 1: Fig. S6B) datasets, respectively. DoubletFinder parameters were optimized using the “paramSweep_v3,” “summarizeSweep,” and “find.pK” functions in the “DoubletFinder” R package, as described previously [34]. DoubletFinder parameters are summarized below:

**Table:** 

Dataset	pK	pN
8-Donor, Lane 1 (A)	0.01	0.25
8-Donor, Lane 2 (A-D)	0.01	0.25
8-Donor, Lane 3 (A-H)	0.01	0.25
8-Donor, Lane 4 (A)	0.01	0.25
Zheng et al., Lane 1 (X)	0.07	0.25
Zheng et al., Lane 2 (Y)	0.09	0.25
Zheng et al., Lane 3 (X,Y)	0.08	0.25

Notably, a simplified quality-control workflow was applied to the 7-donor and 2-condition PBMC datasets to assess the influence of (i) LMO labeling and (ii) SCMK antibody-DNA labeling conditions on SCMK demultiplexing performance. More stringent quality-control steps were not employed because these datasets were not being used to assess alloreactivity gene expression signatures. Briefly, raw gene expression matrices were parsed as described above before the data was log2-transformed, centered, and scaled. Following unsupervised clustering, the top 2000 variable genes (selection.method = “vst”) were then used for dimensionality reduction using PCA and UMAP. Finally, low-quality cells were removed as described above. Summary statistics for each dataset following quality-control are as follows:

**Table:** 

Dataset	nUMI	nGene	nCell
8-Donor PBMC	5042	1265	15,340
Zheng et al. PBMC	1883	681	24,325
7-Donor PBMC	2222	695	25,140
2-Condition PBMC	2836	996	5419

### PBMC cell type annotation

We annotated cell types within each PBMC dataset using literature-supported cell type marker genes [32, 33, 35] and identified most major cell types found in peripheral blood in the 8-donor (Additional file 1: Fig. S5C), Zheng et al. (Additional file 1: Fig. S6C), 7-donor (Additional file 1: Fig. S8A), and 2-condition (Additional file 1: Fig. S8B) PBMC datasets. Marker genes employed are as follows: CD4+ T lymphocytes (IL7R), CD8+ T lymphocytes (CD8A), NK cells (SPON2), B lymphocytes (MS4A1), classical monocytes (CD14), non-classical monocytes (FCGR3A), dendritic cells (CLEC10A), platelets (PF4), proliferative cells (MKI67), plasma cells (MZB1), plasmacytoid dendritic cells (LILRA4), granulocytes (GATA2), neutrophils (LTF), erythrocytes (HBB), macrophages (GBP1), CD3/CD28-stimulated NK cells (GNLY), and CD3/CD28-stimulated T cells (ENO1).

We additionally annotated three CD4+ T cell subsets in the 8-donor (Additional file 1: Fig. S7A), 7-donor (Additional file 1: Fig. S7B), and Zheng et al. (Additional file 1: Fig. S7C) PBMC datasets as follows: activated (SELL-high, S100A4-low, GPR183-high), memory (SELL-low, S100A4-high, GPR183-high), and naïve CD4+ T cells (SELL-high, S100A4-low, GPR183-low).

### MULTI-seq, SCMK, and souporcell classification

For the 8-donor PBMC dataset, cells were classified into donor groups using three different workflows. First, MULTI-seq and SCMK barcode count matrices were fed into the “classifyCells” and “findThresh” functions in the deMULTIplex R package [2]. Second, MULTI-seq and SCMK barcode count matrices and the raw .h5 file (from Cell Ranger) were fed into demuxEM (*p* = 8), an alternative sample classification pipeline [4]. Third, position-sorted BAM files (from Cell Ranger) were fed into the in silico genotyping pipeline, souporcell (*k* = 8) [8]. For the 7-donor PBMC dataset, SCMK barcode count matrices were only analyzed using deMUTIplex, as deMULTIplex, DemuxEM, and souporcell results were observed to be consistent. For the Zheng et al. PBMC dataset, donor identifies were inferred using souporcell (*k* = 2), as MULTI-seq/SCMK barcode count matrices were unavailable. For the 2-condition PBMC dataset, classifications were provided from the supplier.

### PBMC cell type proportion analysis

To determine whether mixing PBMCs from unrelated donors results in changes in PBMC cell type proportions in the 8-donor (Additional file 1: Fig. S3B) and Zheng et al. (Additional file 1: Fig. S4B) PBMC datasets, we first computed the frequency of each cell type grouped according to donor and microfluidic lane. Statistically-significant proportional differences between groups were then identified on a per-cell-type basis using the “pairwise.prop.test” function in the stats R package using default arguments. Evidence of alloreactivity-associated shifts in cell type proportions was assessed by comparing *p* values for donor A cell type proportions. Statistically-significant shifts were never identified between donor A cells from microfluidic lane 1 (A1) and A2/A3 cells, although shifts were detected between A1/A2/A3 and A4, perhaps due to technical variability. This workflow was additionally repeated for CD4+ T cell subsets (8-donor: Additional file 1: Fig. S3D; Zheng et al.: Additional file 1: Fig. S4D) yielding similar results.

### Jensen-Shannon Divergence (JSD) analysis

To perform global comparisons of gene expression profiles between mixed and unmixed PBMCs in the 8-donor and Zheng et al. PBMC datasets, we used JSD in the following workflow. First, each PBMC cell type was randomly down-sampled to include equal numbers of cells from each donor and microfluidic lane (representative UMAPs for 8-donor: Additional file 1: Fig. 3c; Zheng et al.: Additional file 1: Fig. S4E). Down-sampling in this fashion ensures that any observed differences are due to gene expression state and not cell type proportions. Next, UMAP embeddings were computed for each cell type, and UMAP coordinates for each donor/lane group were used to compute group-wise 2-dimensional kernel density estimations with the “kde2d” function in the “MASS” R package [36]. Next, kernel density estimations were fed into the “JSD” function in the philentropy R package [37] to generate a JSD matrix representing the global dissimilarity between each donor/lane group. Finally, JSD scores for each cell type were scaled from 0 to 1, and this process was repeated 100 times. Notably, CD4+ T cells were down-sampled to include equal numbers of each CD4+ T cell subtype from each donor/lane group, and cell types with < 50 cells in any donor/lane group were excluded (e.g., 8-donor: B cells and dendritic cells; Zheng et al.: CD16+ monocytes, dendritic cells, and platelets).

Global differences in gene expression were then summarized as the average and standard deviation of JSD scores across the 100 iterations. Specifically, we quantified the difference between donors (donor A cells from microfluidics lane 1 (A1) vs B2/B3/C2/C3), between mixed and unmixed donor A cells (A1/A4 vs A2/A4), and between technical replicates (A1 vs A4). We then quantified the magnitude of variability due to algorithm performance by repeating this entire workflow after permuting donor A classifications 100 times. Finally, we contextualized the significance of differences in JSD scores associated with mixing status and technical noise via comparison to the average and standard deviation of permuted JSD scores (8-donor: Additional file 1: Fig. 3D; Zheng et al.: Additional file 1: Fig. 4F).

### Gene Set Enrichment Analysis (GSEA)

To perform global comparisons of gene expression profiles between mixed and unmixed PBMCs in the 8-donor and Zheng et al. PBMC datasets, we used GSEA in the following workflow. First, we used the “FindMarkers” differential gene expression analysis function (test.use = “MAST”; logfc.threshold = 0) in Seurat to compute *p* values for every expressed gene among each mixed and unmixed donor A PBMC cell type. Signed *p* values were then fed into GSEA using “pre-ranked” mode, and enriched gene sets were identified as those with nominal *p* values and false discovery rate *q* values below 0.05.

## Supplementary Information

**Additional file 1: Figure S1.** Analysis of SCMK classification bias on PBMC scRNA-seq datasets (e.g., 7-donor PBMC, resting/stimulated PBMC) generated without LMOs. **Figure S2.** Analysis of gene expression signatures associated with Trima apheresis. **Figure S3.** Analysis of PBMC cell type proportions across different experimental conditions. **Figure S4.** Analysis of PBMC cell type proportions and gene expression states linked to alloreactivity in Zheng et al. scRNA-seq dataset. **Figure S5.** scRNA-seq quality-control and cell type annotation workflow, 8-donor PBMC dataset. **Figure S6.** scRNA-seq quality-control and cell type annotation workflow, Zheng et al. PBMC dataset. **Figure S7.** CD4+ T-cell subset classification workflow for 8-donor, 7-donor, and Zheng et al. scRNA-seq datasets. **Figure S8.** PBMC cell type annotation workflow, 7-donor PBMC and resting/stimulated PBMC datasets.

**Additional file 2: Supplemental Table 1.** List of enriched gene sets amongst PBMC cell types as determined by GSEA for 8-donor PBMC scRNA-seq dataset. **Supplemental Table 2.** List of enriched gene sets amongst PBMC cell types as determined by GSEA for Zheng et al. PBMC scRNA-seq dataset.

## Data Availability

All code used for single cell analysis and data visualization is available via Github (github.com/chris-mcginnis-ucsf/PBMC_Allo). Raw gene expression, MULTI-seq, and SCMK barcode count matrices and FASTQs were uploaded to the Gene Expression Omnibus (GSE161329).

## Abbreviations

scRNA-seq: Single-cell RNA sequencing
PBMC: Peripheral blood mononuclear cells
MULTI-seq: Sample multiplexing using lipid-tagged indices
SCMK: Single-cell multiplexing kit
HLA: Human leukocyte antigen
LMO: Lipid-modified oligonucleotide
NK: Natural killer cell
JSD: Jensen-Shannon Divergence
GSEA: Gene Set Enrichment Analysis
