# Supplementary material for: No detectable alloreactive transcriptional responses under standard sample preparation conditions during donor-multiplexed single-cell RNA sequencing of peripheral blood mononuclear cells
Source: BMC Biol. 2021 Jan 20;19:10. doi: 10.1186/s12915-020-00941-x (PMC7816397; doi:10.1186/s12915-020-00941-x)
Supplement: Supplementary file 1 — Additional file 1: Figure S1. Analysis of SCMK classification bias on PBMC scRNA-seq datasets (e.g., 7-donor PBMC, resting/stimulated PBMC) generated without LMOs. Figure S2. Analysis of gene expression signatures associated with Trima apheresis. Figure S3. Analysis of PBMC cell type proportions across different experimental conditions. Figure S4. Analysis of PBMC cell type proportions and gene expression states linked to alloreactivity in Zheng et al. scRNA-seq dataset. Figure S5. scRNA-seq quality-control and cell type annotation workflow, 8-donor PBMC dataset. Figure S6. scRNA-seq quality-control and cell type annotation workflow, Zheng et al. PBMC dataset. Figure S7. CD4+ T-cell subset classification workflow for 8-donor, 7-donor, and Zheng et al. scRNA-seq datasets. Figure S8. PBMC cell type annotation workflow, 7-donor PBMC and resting/stimulated PBMC datasets. [file 12915_2020_941_MOESM1_ESM.pdf]

SUPPLEMENTAL FIGURES

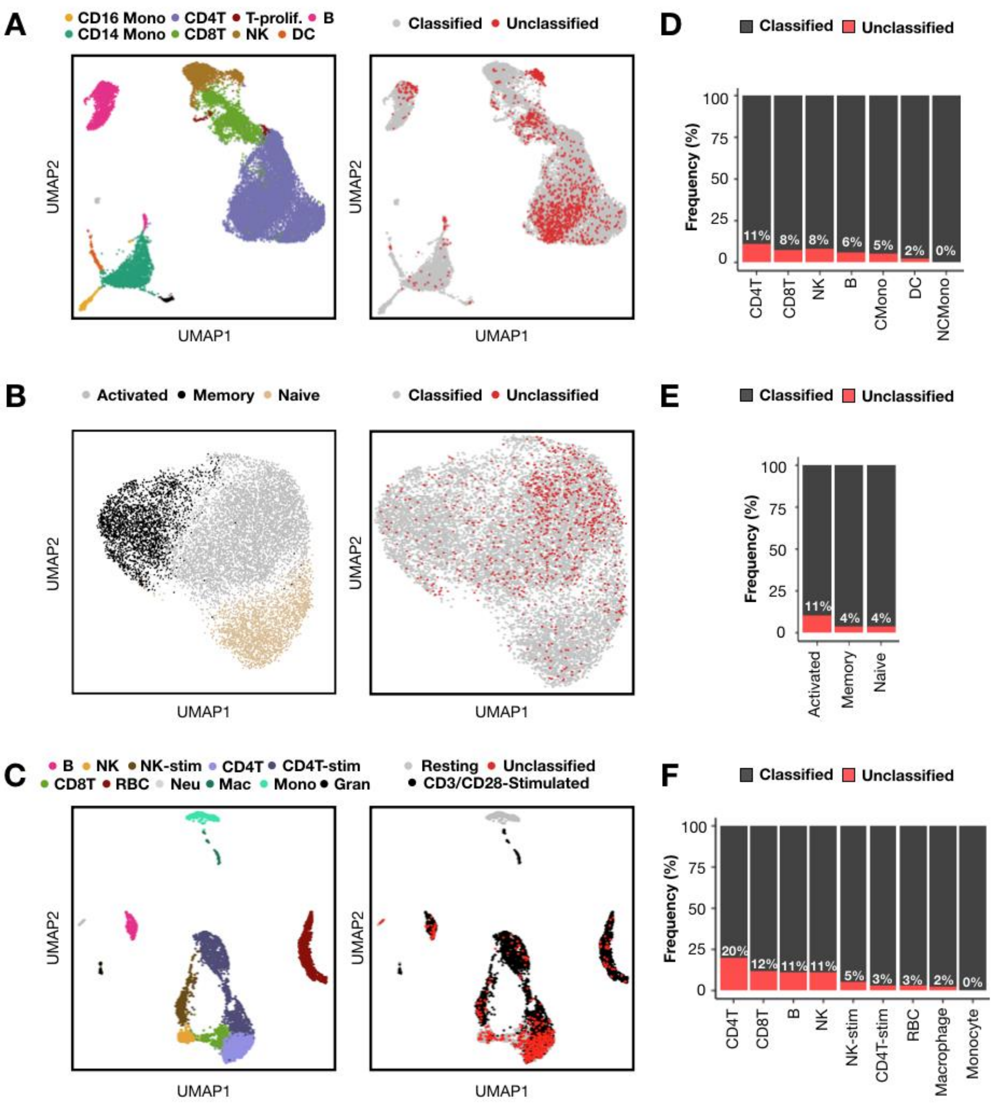

Supplementary Figure S1: Validating SCMK classification biases in scRNA-seq datasets without LMOs

(A-C) Gene expression space colored by cell type annotation (left) and SCMK classification status (right) for PBMCs (A, n = 25,140 cells) and CD4+ T-cells (B; n = 11,297 cells) from a 7-donor multiplexed scRNA-seq experiment, as well as PBMCs from a two-sample (e.g., resting and CD3/CD28-stimulated) scRNA-seq experiment (C, n = 5,419 cells).

(D-F) Frequencies of classified and unclassified cells following SCMK sample demultiplexing across PBMC cell types (D) and CD4+ T-cell subsets (E) from the 7-donor multiplexed scRNA-seq experiment, as well as PBMC cell types from the resting/stimulated scRNA-seq experiment (F).

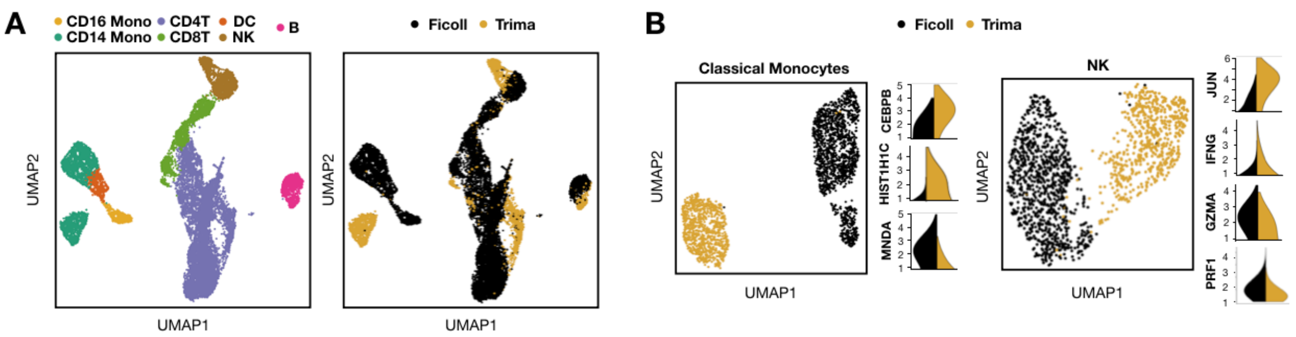

Supplementary Figure S2: Trima-associated gene expression signatures

(A) Identification of gene expression patterns across all PBMC cell-types (left) linked to method of PBMC isolation (e.g., Ficoll or Trima; right). n = 15,340 cells.

(B) Identification of Trima-specific marker genes in classical monocytes (left) and NK cells (right). n = 2,303 classical monocytes, n = 1,545 NK cells. Marker gene expression values are depicted as log1p-normalized counts.

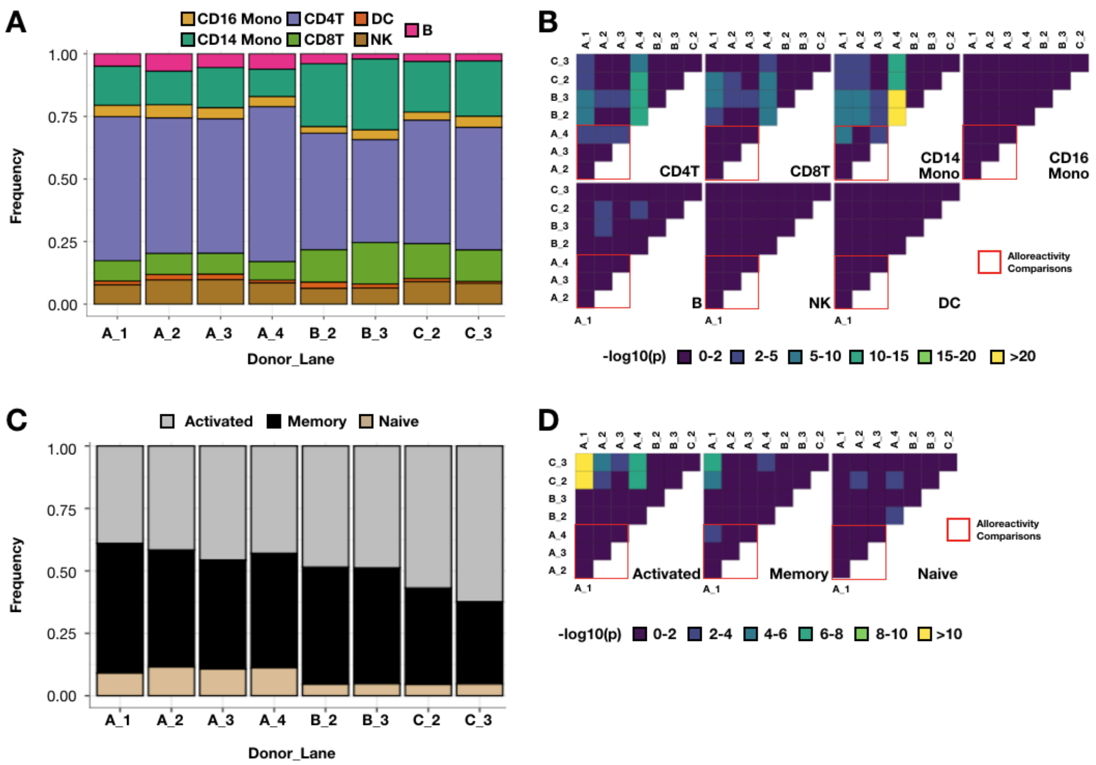

Supplementary Figure S3: PBMC cell type proportions are not influenced by mixing status

(A) PBMC cell-type frequencies for each donor (e.g., A, B, C) and microfluidic lane (e.g., 1, 2, 3, 4).

(B) Pairwise proportion test of PBMC cell type proportion differences between each donor and microfluidic lane, visualized as -log10 p-value heatmaps for each cell type. Red boxes denote key comparisons for assessing putative effects of alloreactivity on cell type proportions. When statistically-significant differences amongst donor A frequencies are detected, they are not linked to mixing status.

(C) CD4+ T-cell subtype frequencies for each donor (e.g., A, B, C) and microfluidic lane (e.g., 1, 2, 3, 4).

(D) Pairwise proportion test of CD4+ T-cell subtype proportion differences between each donor and microfluidic lane, visualized as -log10 p-value heatmaps for each cell type. Red boxes denote key comparisons for assessing putative effects of alloreactivity on subtype proportions. When statistically-significant differences amongst donor A frequencies are detected, they are not linked to mixing status.

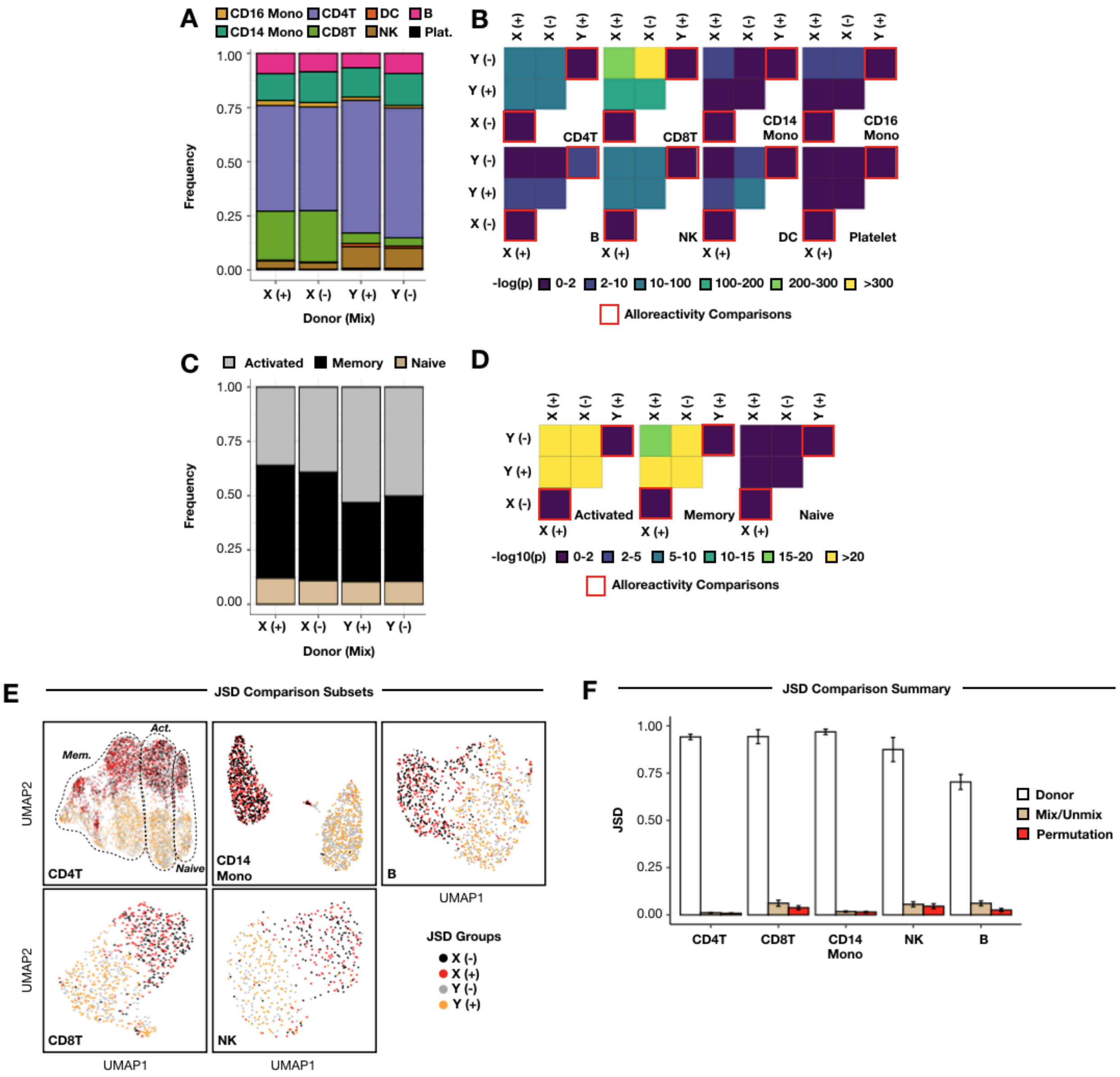

Supplementary Figure S4: No detectable differences in PBMC cell type proportions, CD4+ T-cell proportions, or gene expression state linked to alloreactivity in Zheng et al scRNA-seq data.

(A) PBMC cell-type frequencies grouped by donor (e.g., X, Y) and mixing status (e.g., - = unmixed, + = mixed).

(B) Pairwise proportion test of PBMC cell type proportion differences between mixed and unmixed donors, visualized as -log10 p-value heatmaps for each cell type. Red boxes denote key comparisons for assessing putative effects of alloreactivity on PBMC cell type proportions. Significant differences in cell type proportions are mostly linked to donor, while the singular difference between mixed and unmixed donor Y B-cells is not recapitulated in donor X B-cells.

(C) CD4+ T-cell subtype frequencies grouped as in S4A.

(D) Pairwise proportion test of CD4+ T-cell subtypes proportion differences between mixed and unmixed donors, visualized as in S4B. Significant differences in cell type proportions are entirely linked to donor.

(E) Representative CD4+ T-cell, CD8+ T-cell, CD14+ monocyte, NK cell, and B-cell gene expression embeddings following iterative subsetting to select equal numbers from each JSD comparison group. Cells are colored according to donor ID (e.g., X, Y) and mixing status (e.g., - = unmixed, + = mixed). n = 6,832 CD4+ T-cells, 820 CD8+ T-cells, 1,732 CD14+ monocytes, 488 NK cells, and 1,132 B cells.

(F) JSD analysis summary. Bar plots denote average JSD between PBMC donors (white), mixed/unmixed cells (beige), and cells following label permutation (red). Error bars denote +/- 1 standard deviation. n = 100 iterations.

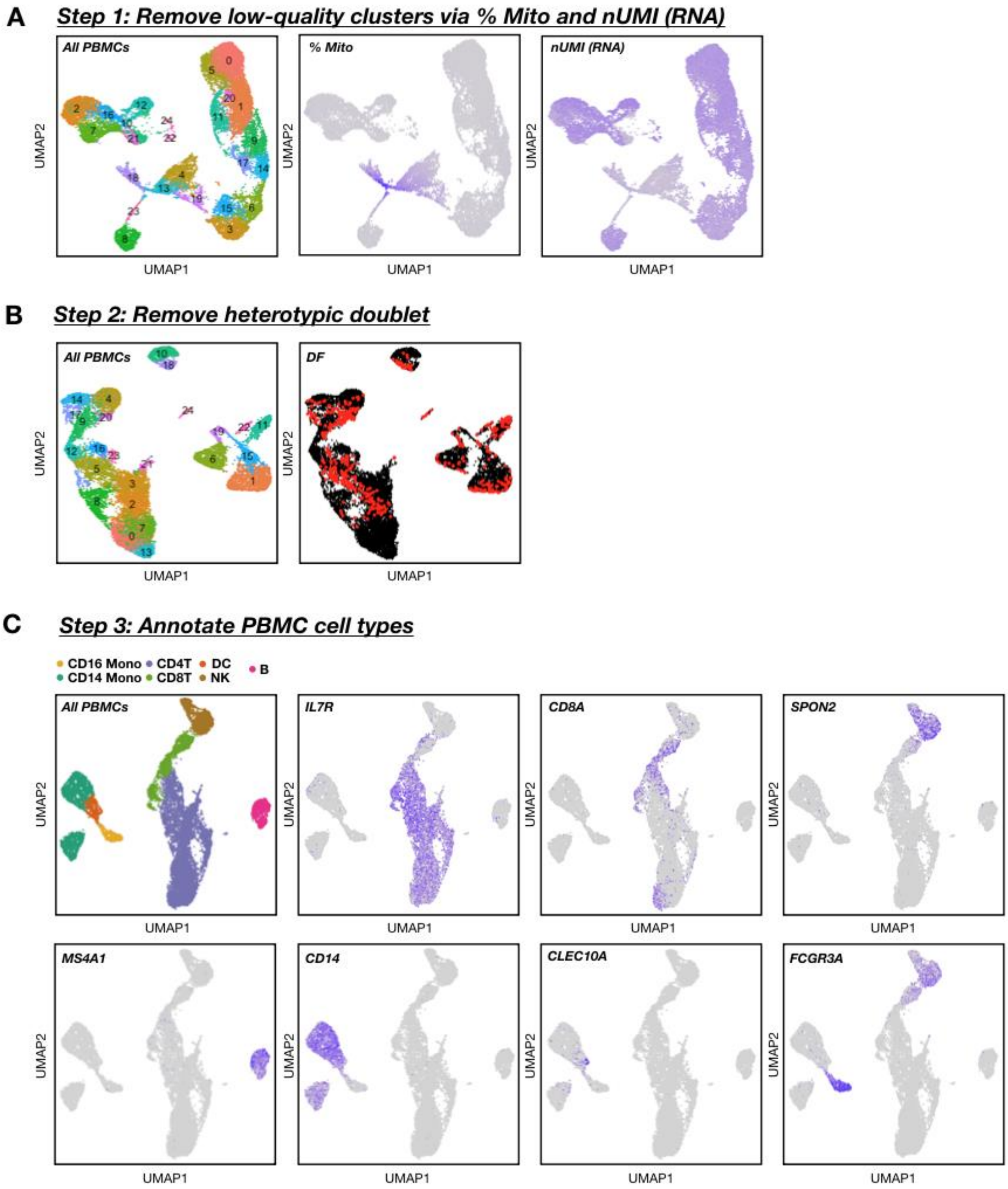

Supplementary Figure S5: scRNA-seq data pre-processing workflow, 8-donor PBMC dataset

(A) Identification of low-quality clusters in gene expression space according to percentage of mitochondrial gene expression (% Mito) and total numbers of RNA UMIs. Clusters 4, 10, 13, 18-20, 23, and 24 were removed. n = 20,353 cells.

(B) Identification of doublets following low-quality cell removal using DoubletFinder (DF). n = 16,627 cells.

(C) Marker genes used for cell type annotations (top left) following low-quality cell and heterotypic doublet removal. CD4+ T-cells (IL7R), CD8+ T-cells (CD8A), NK cells (SPON2), B cells (MS4A1), CD14+ classical monocytes (CD14), dendritic cells (CLEC10A), and CD16+ patrolling monocytes (FCGR3A). n = 15,340 cells

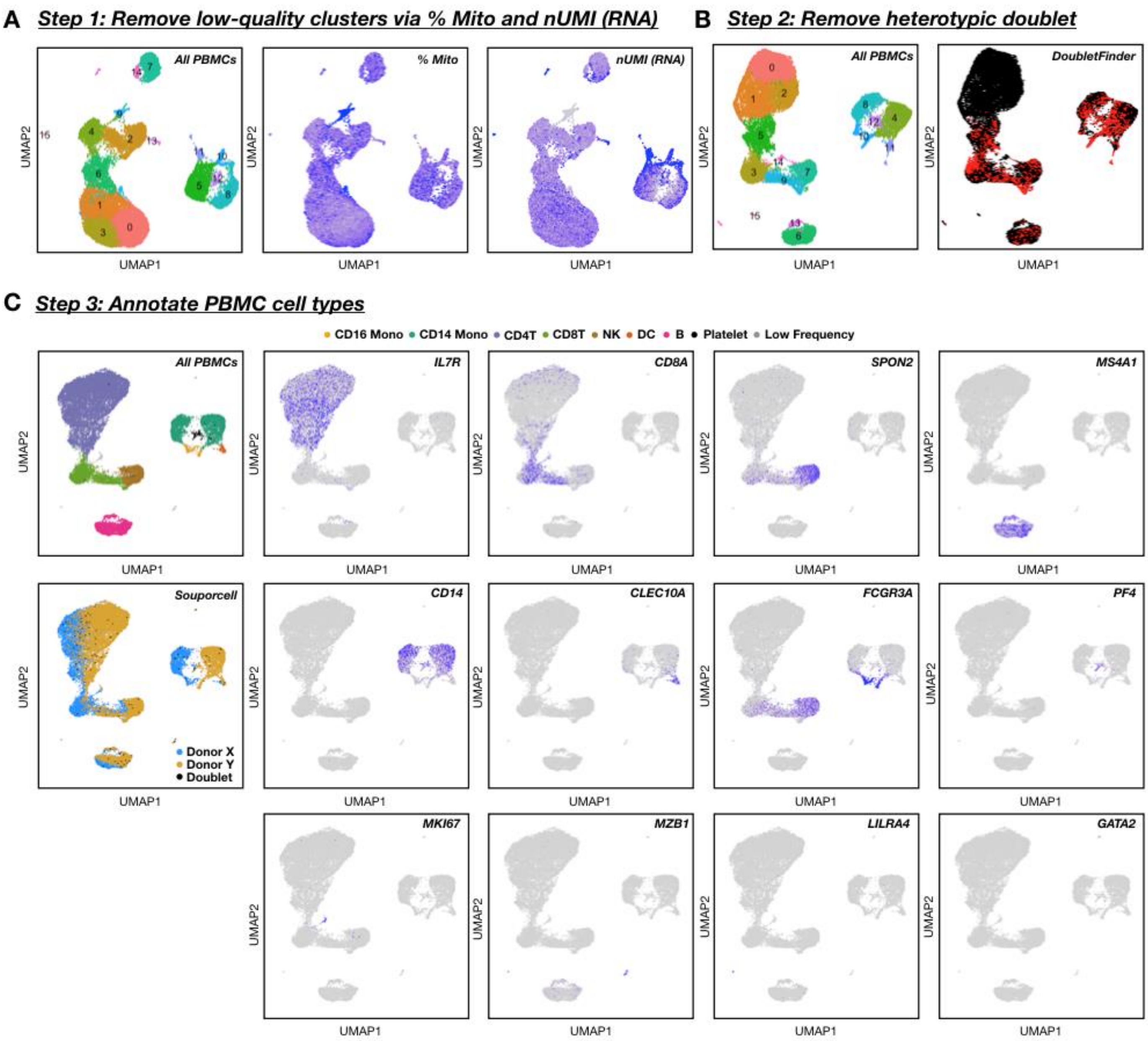

Supplementary Figure S6: scRNA-seq data pre-processing workflow, Zheng et al PBMC dataset

(A) Identification of low-quality clusters in gene expression space according to percentage of mitochondrial gene expression (% Mito) and total numbers of RNA UMIs. Cluster 9 was removed. n = 26,602 cells.

(B) Identification of doublets following low-quality cell removal using DoubletFinder (DF). n = 26,157cells.

(C) Marker genes used for cell type annotations (top left) following low-quality cell and heterotypic doublet removal. CD4+ T-cells (IL7R), CD8+ T-cells (CD8A), NK cells (SPON2), B cells (MS4A1), CD14+ classical monocytes (CD14), dendritic cells (CLEC10A), CD16+ patrolling monocytes (FCGR3A), and platelets (PF4). Low-frequency cell type clusters express markers of proliferation (MKI67), plasma cells (MZB1), plasmacytoid dendritic cells (LILRA4) and granulocytes (GATA2). n = 24,325 cells

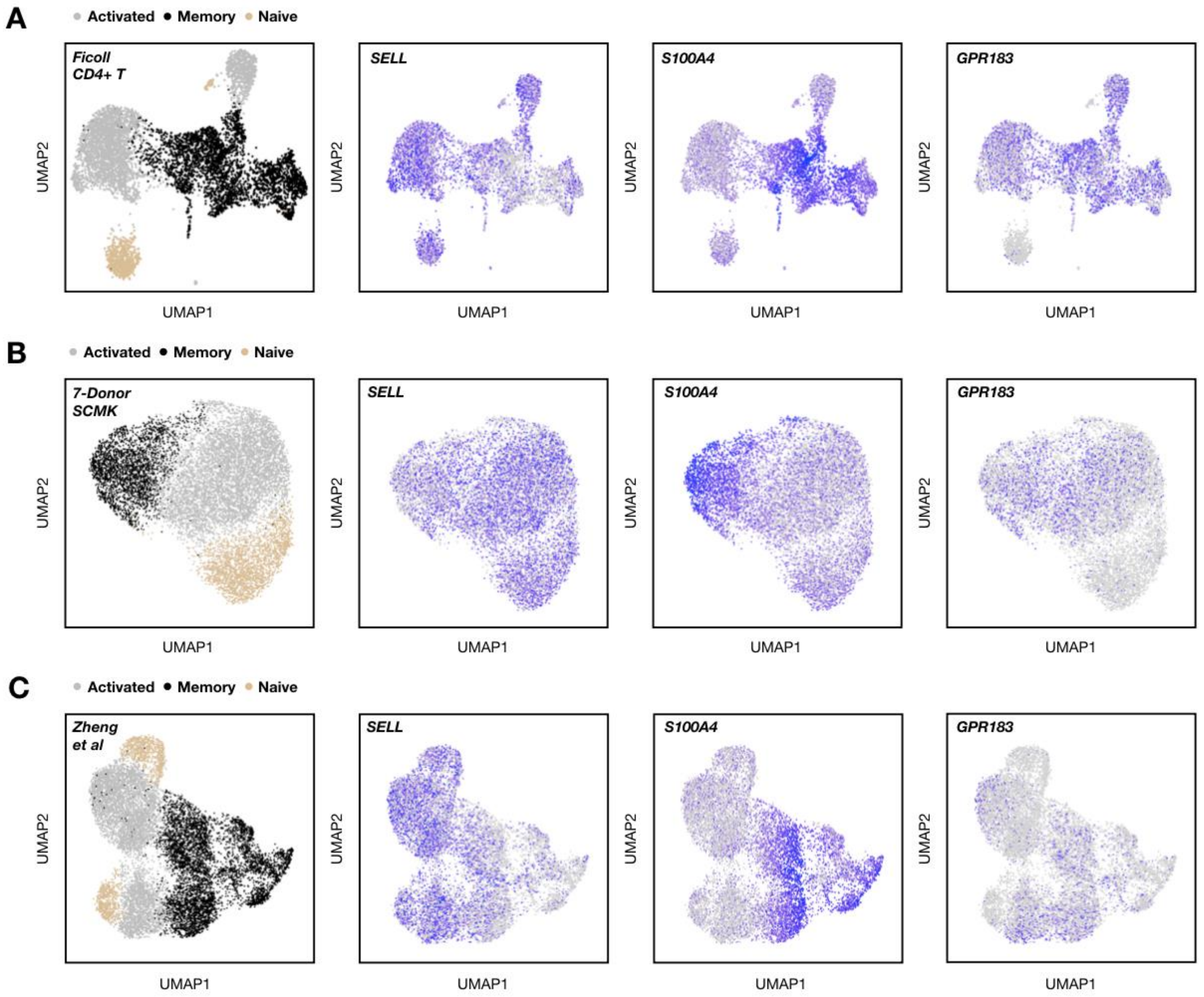

Supplementary Figure S7: T-cell subset annotation strategy

Marker genes used for CD4+ T-cell subtype annotations. Grouped according to SELL/S100A4/GPR183 expression levels, activated CD4+ T-cells are high/low/high, memory CD4+ T-cells are low/high/high, and naive CD4+ T-cells are high/low/low.

(A) Ficoll PBMC CD4+ T-cell gene expression space colored by CD4+ T-cell subset annotation. n= 6,879 cells.

(B) 7-donor SCMK CD4+ T-cell gene expression space colored by CD4+ T-cell subset annotation. n = 11,297 cells.

(C) Zheng et al CD4+ T-cell gene expression space colored by CD4+ T-cell subset annotation. n = 13,166. cells.

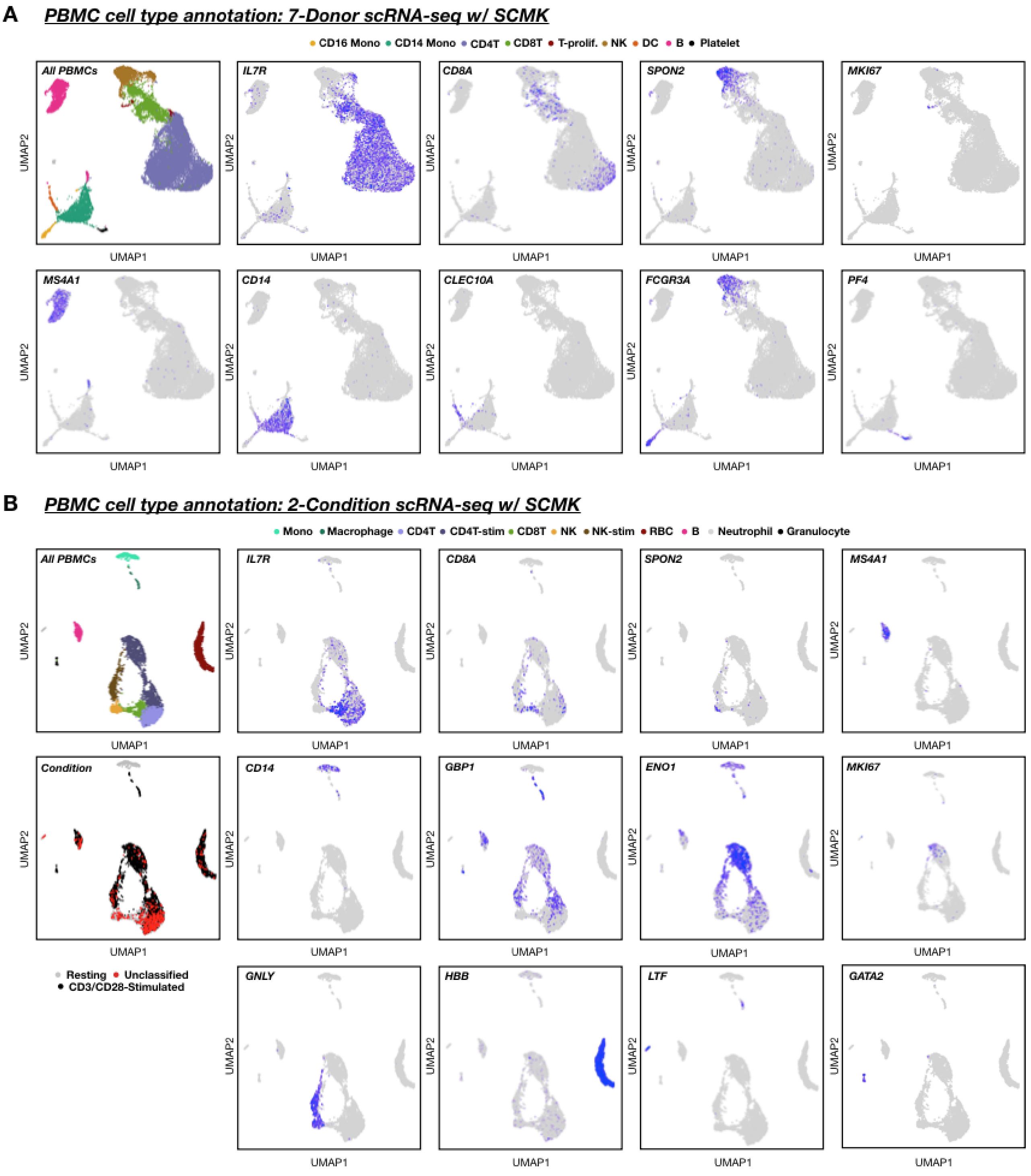

Supplementary Figure S8: Cell type annotation strategy for PBMC scRNA-seq data generated with SCMK

(A) Marker genes used for cell type annotations (top left) for 7-donor SCMK-multiplexed PBMC scRNA-seq data. CD4+ T-cells (IL7R), CD8+ T-cells (CD8A), NK cells (SPON2), proliferating T-cells (MKI67), B cells (MS4A1), CD14+ classical monocytes (CD14), dendritic cells (CLEC10A), CD16+ patrolling monocytes (FCGR3A), and platelets (PF4). n = 25,140 cells.

(B) Marker genes used for cell type annotations (top left) for 2-condition SCMK-multiplexed PBMC scRNA-seq data. CD4+ T-cells (IL7R), CD8+ T-cells (CD8A), NK cells (SPON2), B cells (MS4A1), monocytes (CD14), macrophages (GBP1), stimulated T-cells (ENO1 +/- MKI67), stimulated NK cells (GNLY), erythrocytes (HBB), neutrophils (LTF), and granulocytes (GATA2). n = 5,419 cells.
